# 2′-Hydroxycinnamaldehyde, a Natural Product from Cinnamon, Alleviates Ischemia/Reperfusion-Induced Microvascular Dysfunction and Oxidative Damage in Rats by Upregulating Cytosolic BAG3 and Nrf2/HO-1

**DOI:** 10.3390/ijms252312962

**Published:** 2024-12-02

**Authors:** Yu-Hsuan Cheng, Chih-Yao Chiang, Chung-Hsin Wu, Chiang-Ting Chien

**Affiliations:** 1 School of Life Science, National Taiwan Normal University, Taipei 117, Taiwan; chanel384305@gmail.com; 2 Department of Medicial Research and Division of Cardiovascular Surgery, Cardiovascular Center, Far Eastern Memorial Hospital, New Taipei City 220, Taiwan; eastoneyao@gmail.com; 3 Division of Cardiovascular Surgery, National Defense Medical Center, Taipei 114, Taiwan

**Keywords:** myocardial ischemia/reperfusion injury, 2′-Hydroxycinnamaldehyde, Bcl-2-associated athanogene 3, autophagy, apoptosis, ferroptosis, natural product

## Abstract

2′-Hydroxycinnamaldehyde (HCA), a natural product isolated from the bark of *Cinnamomum cassia*, has anti-inflammatory and anti-tumor activities. In this study, we explored whether HCA preconditioning could protect the heart against ischemia/reperfusion (I/R)-induced oxidative injury through cytosolic Bcl-2-associated athanogene 3 (BAG3) upregulation. In vivo HCA preconditioning was performed intraperitoneally in adult male Wistar rats (50 mg/kg body weight) three times/week for 2 weeks before cardiac I/R injury. The animals were divided into sham control (sham), I/R, and HCA preconditioning plus I/R (HCA+I/R) groups. We examined left ventricular pressure cardiac hemodynamics, the microcirculation, electrocardiograms, infarct size, and oxidative stress and performed Western blots, immunohistochemistry, and cytokine array assays. HCA pretreatment, via BAG3 overexpression, inhibited H_2_O_2_-induced H9c2 cell death. Cardiac I/R injury increased ST-segment elevation, left ventricular end-diastolic pressure, infarct size, myocardial disruption, tissue edema, erythrocyte accumulation, leukocyte infiltration, reactive oxygen species, malondialdehyde, 8-isoprostane, caspase 3-mediated apoptosis, 4HNE/GPX4-mediated ferroptosis, and fibrosis but decreased the microcirculation, cytosolic BAG3, and Beclin-1/LC3 II-mediated autophagy in the I/R hearts. HCA preconditioning significantly decreased these oxidative injuries by increasing cardiac cytosolic BAG3 and Nrf2/HO-1 signaling. HCA preconditioning significantly decreased cardiac I/R-enhanced mitochondrial fission DRP1 expression. Our data suggest that HCA preconditioning can efficiently improve myocardial I/R injury-induced cardiac dysfunction, apoptosis, ferroptosis, mitochondrial fission, and autophagy inhibition through cardiac BAG3 and Nrf2/HO-1 upregulation.

## 1. Introduction

Coronary artery disease, also known as ischemic heart disease, impairs cardiovascular function by increasing myocyte rupture, vascular endothelial cell aggregation and stagnation, and leukocyte infiltration and adhesion, subsequently leading to acute myocardial infarction (AMI) [1,2]. Clinical trials frequently use thrombolytic therapy or percutaneous coronary intervention for the timely reperfusion of ischemic tissue to reduce infarct size [3,4]. However, myocardial reperfusion can further induce and exacerbate myocardial cell damage, a phenomenon commonly referred to as myocardial ischemia/reperfusion (I/R) injury [5,6,7,8]. Myocardial I/R injury involves many mechanisms, such as the inflammatory response, oxidative stress, Ca^2+^ overload, ferroptosis, endoplasmic reticulum stress, autophagy, and energy metabolism disorders, and its complex pathogenic mechanisms have not been fully elucidated [9]. Therefore, efforts are underway to elucidate the exact mechanism of myocardial I/R injury in order to develop treatment strategies to minimize its consequences. It is well known that mitochondrial dysfunction and impairment play critical roles in cardiac I/R injury through exacerbated reactive oxygen species (ROS) production and oxidative stress, consequently leading to several types of programmed cell death, including apoptosis, ferroptosis, and autophagy [9,10,11]. The pathologic mechanisms are possibly due to the formation of H_2_O_2_, O_2_-, and OH, leading to lipid peroxidation, protein oxidation, and DNA damage-induced cardiovascular diseases [12,13]. Therefore, cumulative evidence has indicated that cardiac I/R-induced myocardial damage is closely correlated with mitochondrial impairment, cardiomyocyte apoptosis, ferroptosis, and oxidative stress [9,10,11,12,13].

Bcl-2-associated athanogene 3 (BAG3), a member of the BAG family of proteins, can regulate apoptosis, autophagy, and development to help cells adapt to oxidative stress [14]. BAG3 is highly expressed in the heart, maintains the integrity of the sarcomere structure and mitochondria, and reduces cardiomyocyte apoptosis [15]. Cumulative evidence shows that decreases in BAG3 expression cause myofibrillar disorders and severe left ventricular dysfunction, a decrease in ejection fraction [16], mitochondrial structure alterations [17], excess ROS production [10,11], a decrease in autophagy, and an increase in apoptosis [15]. It has also been found that upregulation of the BAG3 protein can improve the cardiac function of mice with I/R-induced myocardial infarction and reduce cardiomyocyte death [18]. BAG3 can induce vasodilation and activate PI3K/AKT/eNOS signaling to enhance endothelial function [19]. BAG3, via autophagy and lysosomal pathways, clears damaged mitochondria [20], providing a new therapeutic target in cardiac I/R injury [14]. Nuclear factor erythroid 2-related factor (Nrf2) is another critical antioxidant transcription factor, and it regulates the expression of multiple genes in the process of ferroptosis, such as glutathione peroxide 4 (GPX4) [21].

Cinnamaldehyde is a bioactive component isolated from the Chinese herbal medicine *Cinnamonum cassia*, and it has many biomedical functions [22,23,24,25,26,27,28,29]. 2′-Hydroxycinnamaldehyde (HCA), a natural derivative of cinnamaldehyde, can exert anti-tumor activity [30,31]. In addition, HCA upregulates BAG3 expression and mediates apoptosis in cancer cells [32]. Cinnamaldehyde may exert antioxidant and anti-inflammatory action to reduce cardiac I/R injury [33] and activate Nrf2/heme oxygenase-1 (HO-1) signaling to protect human endothelial cells against oxidative stress-induced damage [34]. Based on these findings, HCA may be an important component of a therapeutic strategy to ameliorate oxidative stress-induced cardiovascular diseases. However, no such evidence has been reported. The present study was designed to investigate the protective effect and mechanisms of HCA preconditioning in preventing and attenuating cardiac I/R injury in vitro and in vivo.

## 2. Results

### 2.1. HCA Preconditioning Inhibited H_2_O_2_-Induced Cell Death and Upregulated BAG3 Expression in H9c2 Cells

**Figure 1:**
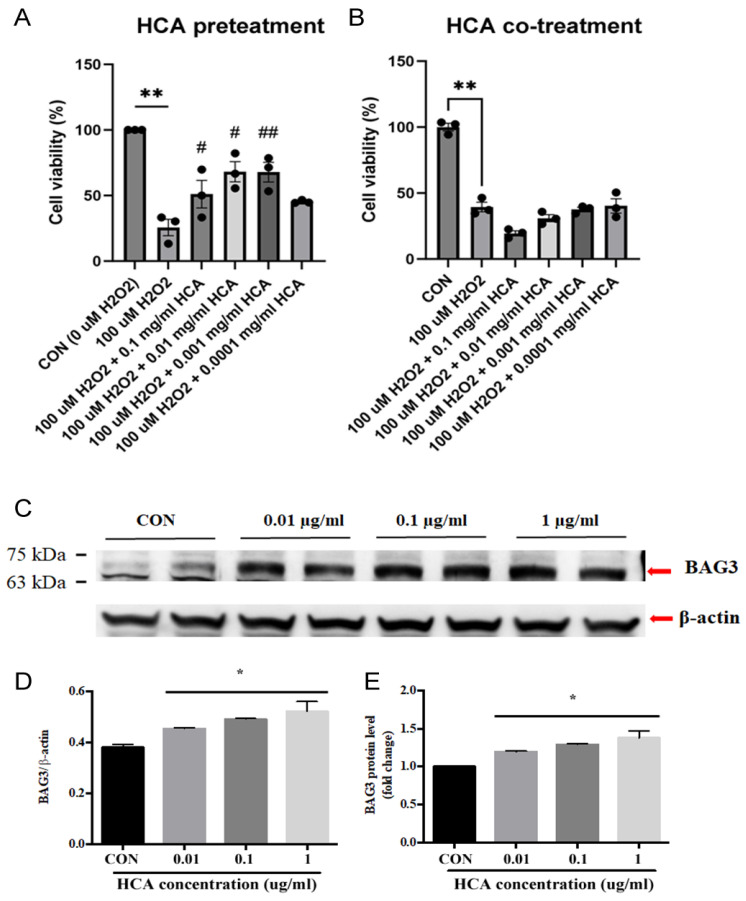
Effect of HCA pretreatment on H_2_O_2_-induced H9c2 cell death and BAG3 expression in H9c2 cells. HCA pretreatment (**A**) but not co-treatment (**B**) significantly inhibited H_2_O_2_-induced H9c2 cell death at a dose of 0.001–0.1 mg/mL. HCA pretreatment induced a dose-dependent upregulation of BAG3 expression in H9c2 cells (**C**–**E**). All data are expressed as mean ± SEM (n = 3). * indicates significance vs. CON group (* *p* < 0.05; ** *p* < 0.01). # indicates significance vs. H_2_O_2_ group (# *p* < 0.05; ## *p* < 0.01).

As shown in Figure 1A,B, HCA pretreatment significantly inhibited H_2_O_2_-induced H9c2 cell death in a dose-dependent manner, whereas HCA co-treatment did not exert a protective effect on H_2_O_2_-induced H9c2 cell death. These data suggest that cellular protection may come from a later protection mechanism. We further examined the effects of HCA preconditioning on BAG3 expression in H9c2 cells. As shown in Figure 1C, our data demonstrate that HCA preconditioning significantly enhanced BAG3 expression in H9c2 cells in a dose-dependent manner, with 0.1 mg/mL of HCA achieving the maximal enhancement in BAG3 expression (Figure 1D,E).

### 2.2. HCA Preconditioning Increased Cytosolic but Not Mitochondrial BAG3 Expression in HCA and HCA+I/R Hearts

**Figure 2:**
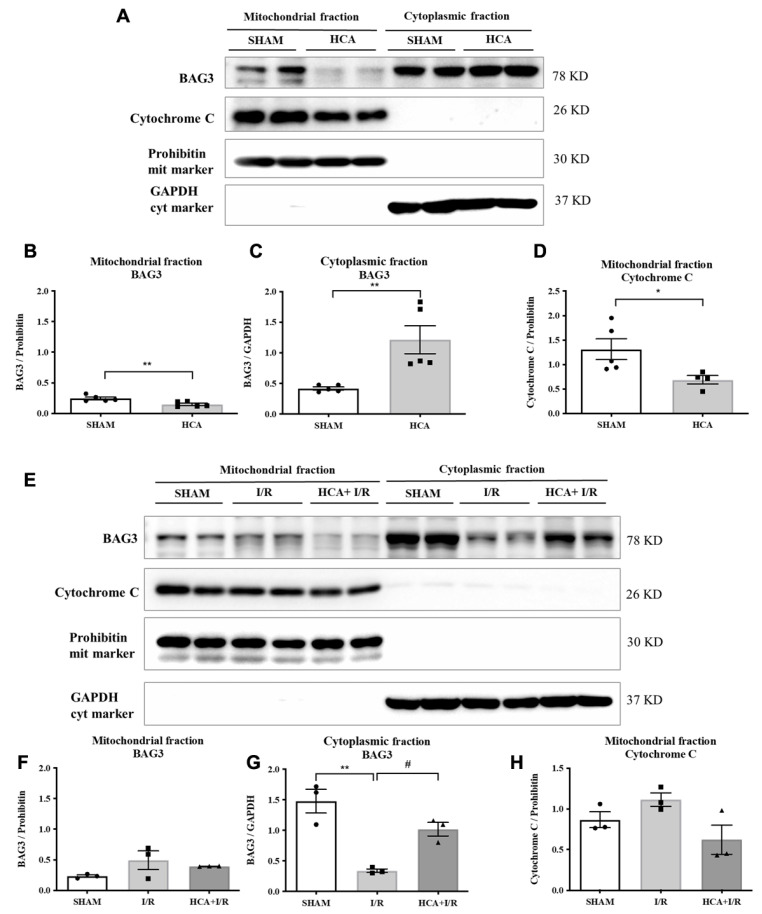
Effect of HCA preconditioning on mitochondrial and cytosolic BAG3 expression in sham, HCA, I/R, and HCA+I/R groups. (**A**) Original graphs of cytosolic and mitochondrial BAG3 expression in sham and HCA hearts, determined via Western blot assay. Translocation of mitochondrial BAG3 (**B**) to cytosolic BAG3 (**C**) and translocation of mitochondrial cytochrome C (**D**) are noted between sham and HCA hearts. (**E**) Original traces of mitochondrial and cytosolic BAG3 expression. Significantly reduced cytosolic BAG3 expression is observed in I/R group vs. sham group, whereas significantly preserved cytosolic BAG3 is observed in HCA+I/R group vs. I/R group (**G**). Mitochondrial fractions of BAG3 expression (**F**) and cytochrome C expression (**H**) are not significantly different among sham, I/R, and HCA+I/R groups. Each symbol represents the respective individual. All data are expressed as mean ± SEM (n = 3–5). * indicates significance vs. sham group (* *p* < 0.05; ** *p* < 0.01). # indicates significance vs. I/R group (# *p* < 0.05).

Figure 2A presents original graphs of cytosolic and mitochondrial BAG3 expression in the sham and HCA hearts, determined via a Western blot assay. Decreased mitochondrial BAG3 expression (Figure 2B), increased cytosolic BAG3 expression (Figure 2C), and decreased mitochondrial cytochrome C (Figure 2D) were noted between the sham and HCA hearts. Figure 2E presents the original traces of mitochondrial and cytosolic BAG3 expression among the sham, I/R, and HCA+I/R hearts. Significantly reduced cytosolic BAG3 expression was found in the I/R group vs. the sham group, whereas significantly preserved cytosolic BAG3 was observed in the HCA+I/R group vs. the I/R group (Figure 2G). The mitochondrial fractions of BAG3 expression (Figure 2F) and cytochrome C expression (Figure 2H) were not significantly different among the sham, I/R, and HCA+I/R groups.

### 2.3. HCA Preconditioning Preserved Cardiac I/R-Induced Decrease in Cardiac Surface Microcirculation

**Figure 3:**
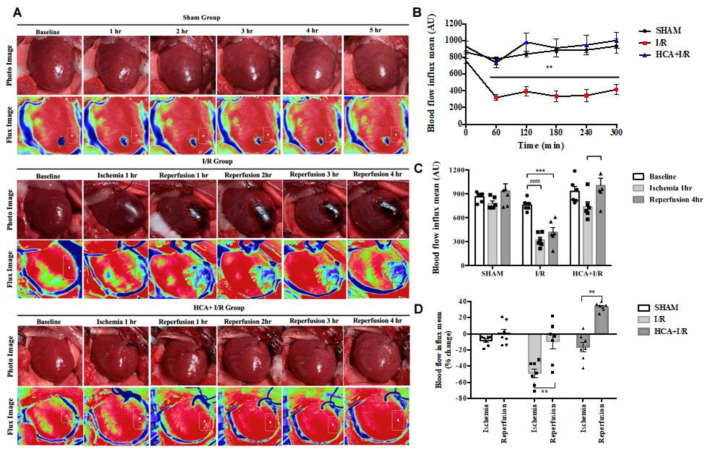
Effect of HCA preconditioning on cardiac I/R-induced changes in cardiac surface microcirculation. (**A**) Representative responses of cardiac microcirculation to cardiac I/R in the three groups of rats. (**B**) Statistical data of cardiac surface microcirculation. Significant decrease in cardiac microcirculation is observed in I/R group vs. sham group, whereas cardiac microcirculation is restored in HCA+I/R group. (**C**) Cardiac surface blood flow responses to baseline, 1 h of ischemia, and 4 h of reperfusion among the three groups. (**D**) Mean percentage change in cardiac surface blood flow during ischemia and reperfusion among the three groups. All data are expressed as mean ± SEM (n = 6). * indicates significance vs. sham group (** *p* < 0.01; *** *p* < 0.001). # indicates significance vs. I/R group (#### *p* < 0.0001).

After the anesthetized rats underwent thoracotomy to expose the heart, the cardiac surface blood flow was continuously monitored in the three groups via laser speckle imaging at different time points (Figure 3A). Cardiac I/R significantly reduced the blood flow intensity in the I/R group vs. the sham group (Figure 3B). However, HCA preconditioning significantly preserved the cardiac surface microcirculation in the HCA+I/R hearts vs. the I/R hearts. The decreased percentage of blood flow intensity during ischemia or reperfusion in the I/R group was significantly restored in the HCA+I/R group (Figure 3C,D). There was no significant drop in blood flow after ischemia, but there was a tendency for the blood flow to increase after reperfusion, demonstrating that HCA preconditioning could protect against the phenomenon of ischemic death due to cardiac I/R.

### 2.4. HCA Preconditioning Restored Cardiac I/R-Induced Increase in LVEDP and Contractile Activity

**Figure 4:**
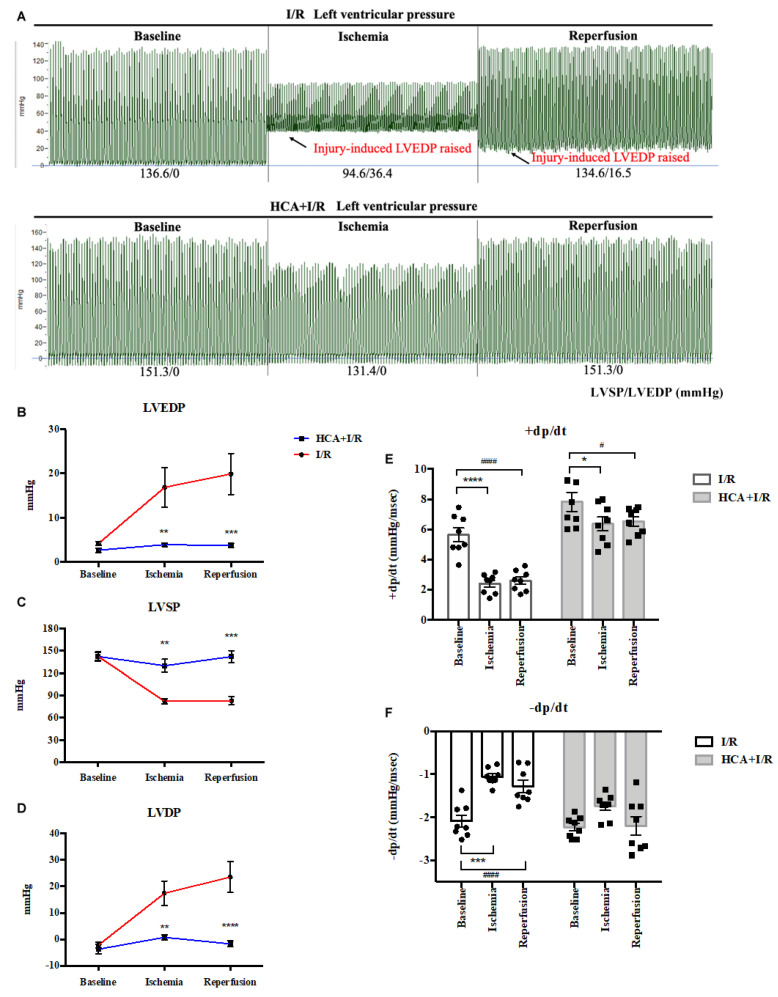
Effect of HCA preconditioning on left ventricular pressure in I/R and HCA+I/R hearts. (**A**) Typical responses of left ventricular pressure during baseline, ischemia, and reperfusion phases in two groups. Statistical data for LVEDP (**B**), LVSP (**C**), LVDP (**D**), +dp/dt (**E**), and -dp/dt (**F**) between I/R and HCA+I/R groups. All data are expressed as mean ± SEM (n = 8). * indicates significance vs. sham group (* *p* < 0.05; ** *p* < 0.01; *** *p* < 0.001; **** *p* < 0.0001). # indicates significance vs. I/R group (# *p* < 0.05; #### *p* < 0.0001).

The responses of left ventricular pressure during I/R periods in the I/R and HCA+I/R groups are shown in Figure 4A. The statistical data indicate that cardiac I/R significantly increased LVEDP (Figure 4B) and LVDP (Figure 4D) and that it decreased LVSP (Figure 4C) and ±dp/dt (Figure 4E,F) in the I/R group vs. the HCA+I/R group. HCA preconditioning can effectively improve the strength of myocardial contraction and diastole, maintain the function of cardiac diastole and contraction, and effectively improve the dysfunction of cardiac diastolic and systolic function caused by cardiac I/R injury.

### 2.5. HCA Preconditioning Improved Cardiac I/R-Altered EKG Parameters

**Figure 5:**
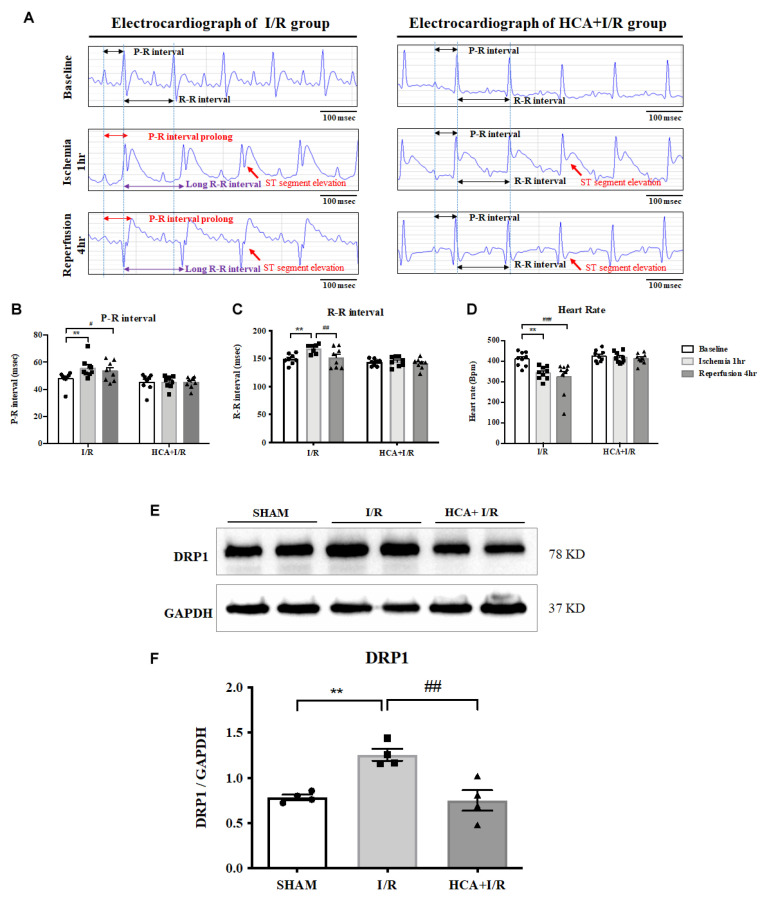
Effect of HCA preconditioning on cardiac I/R-affected EKG parameters and mitochondrial fission. (**A**) Representative EKG graphs in ST segment, with P-R and R-R intervals, in I/R and HCA+I/R groups. Statistical data for P-R intervals (**B**), R-R intervals (**C**), and heart rate (**D**). Expression of the mitochondrial fission marker DRP1 protein (**E**) in the three groups and statistical data (**F**). All data are expressed as mean ± SEM (n = 8). * indicates significance vs. sham group (** *p* < 0.01). # indicates significance vs. I/R group (# *p* < 0.05; ## *p* < 0.01; ### *p* < 0.001).

We used iWorx 214 to determine EKG parameters, including the ST segment, R-R and P-R intervals, and heart rate, in the I/R and HCA+I/R groups (Figure 5A). Cardiac I/R markedly enhanced ST-segment elevation in both the I/R and HCA+I/R groups, indicating the successful induction of AMI. The statistical data show that a significant increase in the P-R (Figure 5B) and R-R intervals (Figure 5C) and a significant decrease in heart rate (Figure 5D) occurred in the I/R period vs. the baseline stage. HCA preconditioning significantly restored these altered parameters in the HCA+I/R group vs. the I/R group, indicating its ability to maintain the rhythmic function of the heart.

### 2.6. Effects of Bioactive Peptides from Tortoiseshell, Antler, and Their Combination on the Cell Viability of MC3T3-E1 Osteoblasts and HIG-82 Chondrocytes

Cardiac I/R significantly increased the expression of the mitochondrial fission marker DRP1 in the I/R group vs. the sham group; however, HCA preconditioning significantly decreased the cardiac I/R-induced increase in DRP1 expression in the HCA+I/R group vs. the I/R group (Figure 5E,F).

### 2.7. HCA Preconditioning Obviously Improved Cardiac I/R-Induced Pathologic Alterations

**Figure 6:**
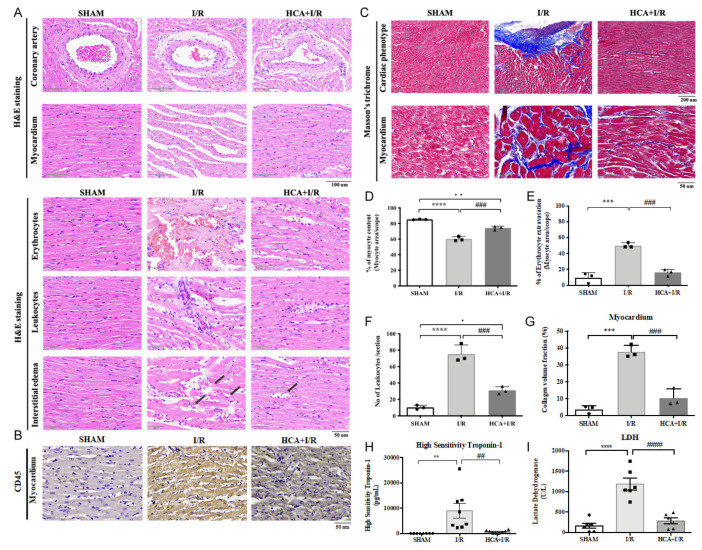
Effect of HCA preconditioning on cardiac I/R-induced pathologic changes, leukocyte infiltration, and fibrosis among sham, I/R, and HCA+I/R groups. (**A**) Structural alterations in response to cardiac I/R are demonstrated among the three groups via H&E staining. (**B**) Immunohistochemistry of CD45 staining (leukocyte biomarker) in the three groups. (**C**) Degree of fibrosis in heart tissue after cardiac I/R injury is indicated using Masson’s trichrome staining of the three groups. Percentage of myocyte content (**D**), percentage of erythrocyte extravasation (**E**), leukocyte infiltration (**F**), collagen volume fraction (**G**), high-sensitivity troponin-1 (**H**), and lactate dehydrogenase level (**I**) are compared among the three groups. All data are expressed as mean ± SEM. * indicates significance vs. sham group (** *p* < 0.01; *** *p* < 0.001; **** *p* < 0.0001). # indicates significance vs. I/R group (## *p* < 0.01; ### *p* < 0.001; #### *p* < 0.0001).

In the histological examination of the H&E-stained heart tissues, cardiac I/R injury caused the tightly arranged myocardial tissue to become a fragmented structure and obvious interstitial edema (Figure 6A). Excessive erythrocyte extravasation and leukocyte infiltration were also observed in the I/R tissues (Figure 6A). A specific biomarker of CD45 for leukocyte staining significantly increased in the I/R vs. HCA+I/R groups (Figure 6B). The coronary arteries and the surrounding tissues also showed a pathological phenomenon of detachment and damage (Figure 6A). Compared with the I/R group, the above symptoms significantly improved in the HCA+I/R group (Figure 6D–F). Therefore, HCA preconditioning can significantly improve and reduce the damage to and destruction of the cardiac tissue structure caused by cardiac I/R.

### 2.8. HCA Preconditioning Decreased Cardiac I/R-Induced Fibrosis

Masson’s trichrome staining indicated that cardiac I/R markedly enhanced blue-stain collagen fiber accumulation after I/R injury (Figure 6C), whereas HCA preconditioning significantly decreased the degree of fibrosis by collagen content vs. the I/R hearts (Figure 6G).

### 2.9. HCA Preconditioning Attenuated Cardiac I/R-Induced Increase in cTn I and LDH Levels

The plasma levels of high-sensitivity troponin-1 (cTn I) and lactate dehydrogenase (LDH) were used as biomarkers for AMI prognosis. Our results show that the cTn I and LDH values were significantly increased in the I/R group vs. the sham group, whereas they were significantly decreased in the HCA+I/R group vs. the I/R group (Figure 6H,I).

### 2.10. HCA Preconditioning Decreased Cardiac I/R-Induced Infarct Size

**Figure 7:**
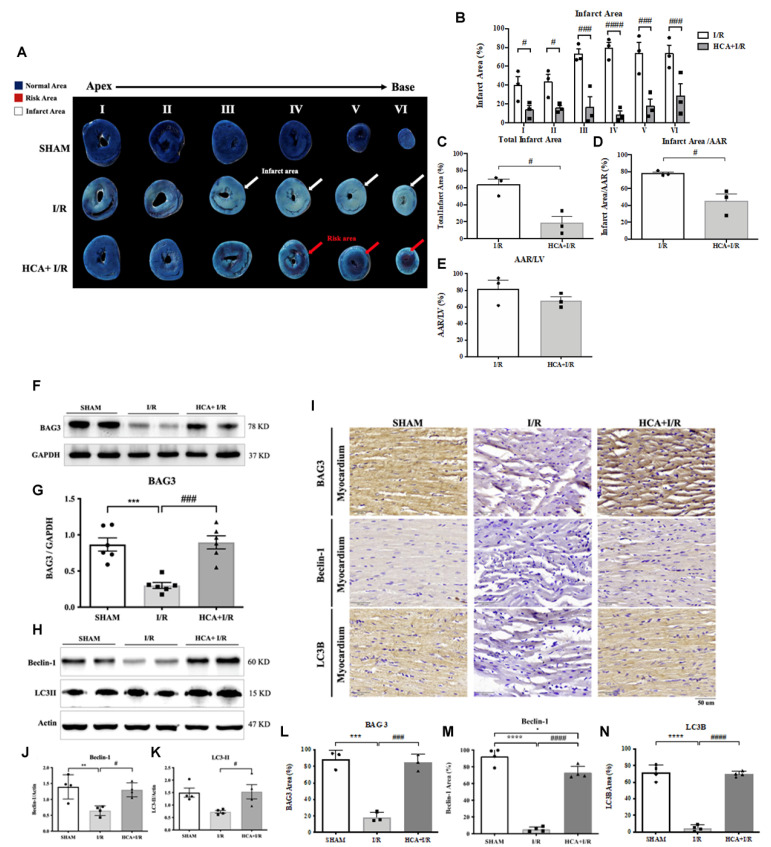
Effect of HCA preconditioning on cardiac I/R-induced infarct size, BAG3, and autophagy expression among the three groups. The typical infarct areas of I-VI sections among the three groups are displayed in (**A**). Percentages of six sections of infarct area in I/R and HCA+I/R groups are indicated in (**B**). Total infarct area (**C**), infarct area/AAR (**D**), and AAR/LV (**E**) are demonstrated. Cardiac BAG3 expression (**F**,**G**), Beclin-1 (**H**,**J**), and LC3II (**H**,**K**) examined via Western blot analysis are also shown. Immunohistochemistry of BAG3, Beclin-1, and LC3II is shown in (**I**). Statistical analysis of BAG3, Beclin-1, and LC3II in the three groups is shown in (**L**–**N**), respectively. All data are expressed as mean ± SEM (n = 3). * indicates significant differences vs. sham group (** *p* < 0.01; *** *p* < 0.001; **** *p* < 0.0001). # indicates significant differences vs. I/R group (# *p* < 0.05; ### *p* < 0.001; #### *p* < 0.0001).

By using Evans blue-TTC double staining, we characterized the cardiac I/R-induced infarct area, area at risk, and total left ventricular area. Figure 7A shows representative images of the infarct size in six sections in the three groups. Cardiac I/R significantly increased the infarct area (Figure 7B), total infarct area (Figure 7C), and infarct area/AAR (Figure 7D) in the I/R hearts vs. the sham hearts, whereas HCA preconditioning significantly reduced these parameters in the HCA+I/R group vs. the I/R group.

### 2.11. HCA Preconditioning Enhanced BAG3 Expression and Restored Autophagy-Related Proteins in I/R Hearts

Cardiac I/R significantly decreased cardiac BAG3 expression in the I/R vs. sham group (Figure 7F,G), whereas HCA preconditioning efficiently preserved BAG3 expression in the HCA+I/R vs. I/R group. I/R significantly inhibited cardiac Beclin-1 (Figure 7H,J) and LC3II expression (Figure 7H,K) in the I/R vs. sham group; however, HCA preconditioning significantly restored Beclin-1 and LC3II in the HCA+I/R vs. I/R group. Typical immunohistochemistry of cardiac BAG3, Beclin-1, and LC3II expression is demonstrated in Figure 7I. The statistical data for the immunohistochemistry of BAG3, Beclin-1, and LC3II in the three groups are consistent with the Western blot and are presented in Figure 7L, M, and N, respectively.

### 2.12. HCA Preconditioning Decreased Cardiac I/R-Induced 4HNE/GPX4-Mediated Ferroptosis and Caspase 3-Mediated Apoptosis

**Figure 8:**
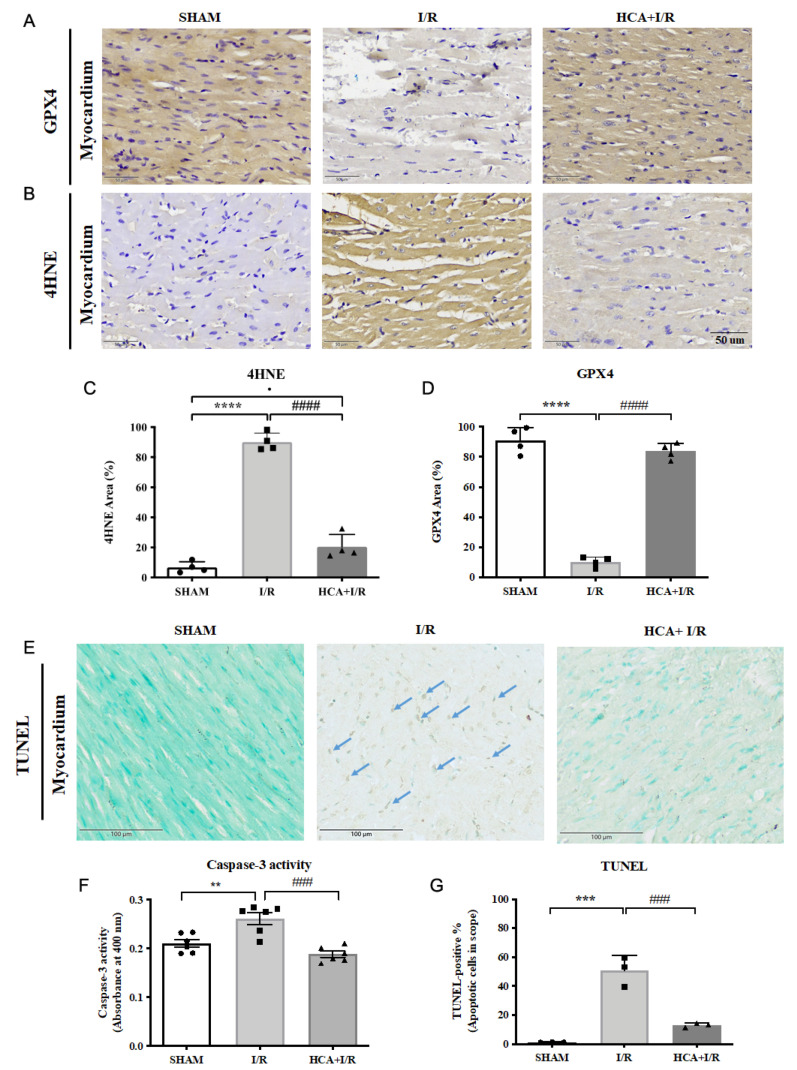
Effect of HCA preconditioning on cardiac I/R-induced 4HNE/GPX4-mediated ferroptosis and caspase 3-mediated apoptosis in the three groups. Typical GPX4 and 4HNE stains of the three groups are displayed in (**A**,**B**), respectively. The percentages of staining of 4HNE and GPX4 sections in the three groups are indicated in (**C**,**D**). Cardiac TUNEL expression in the three groups is shown in (**E**). Statistical analysis of caspase 3 activity is presented in (**F**). Statistical analysis of TUNEL-positive cells in the three groups is presented in (**G**). All data are expressed as mean ± SEM (n = 3). * indicates significant differences vs. sham group (** *p* < 0.01; *** *p* < 0.001; **** *p* < 0.0001). # indicates significant differences vs. I/R group (### *p* < 0.001; #### *p* < 0.0001).

Typical GPX4 and 4HNE stains of the three groups are displayed in Figure 8A,B, respectively. Cardiac I/R significantly enhanced the percentage of 4HNE staining (Figure 8C) and significantly decreased GPX4 staining (Figure 8D) vs. the sham group. Furthermore, cardiac I/R markedly increased TUNEL-positive stained cells in the I/R hearts vs. the sham group (Figure 8E). A statistical analysis of caspase 3 activity (Figure 8F) and TUNEL-positive cells showed that they significantly increased (Figure 8G) in the I/R group vs. the sham group. HCA preconditioning significantly decreased 4HNE staining (Figure 8C) and preserved GPX4 staining (Figure 8D) in the HCA+I/R vs. I/R group. HCA preconditioning significantly decreased caspase 3 activity (Figure 8F) and TUNEL-positive apoptosis staining (Figure 8G) in the HCA+I/R vs. I/R group.

### 2.13. HCA Provided Antioxidant Activity In Vitro and In Vivo

**Figure 9:**
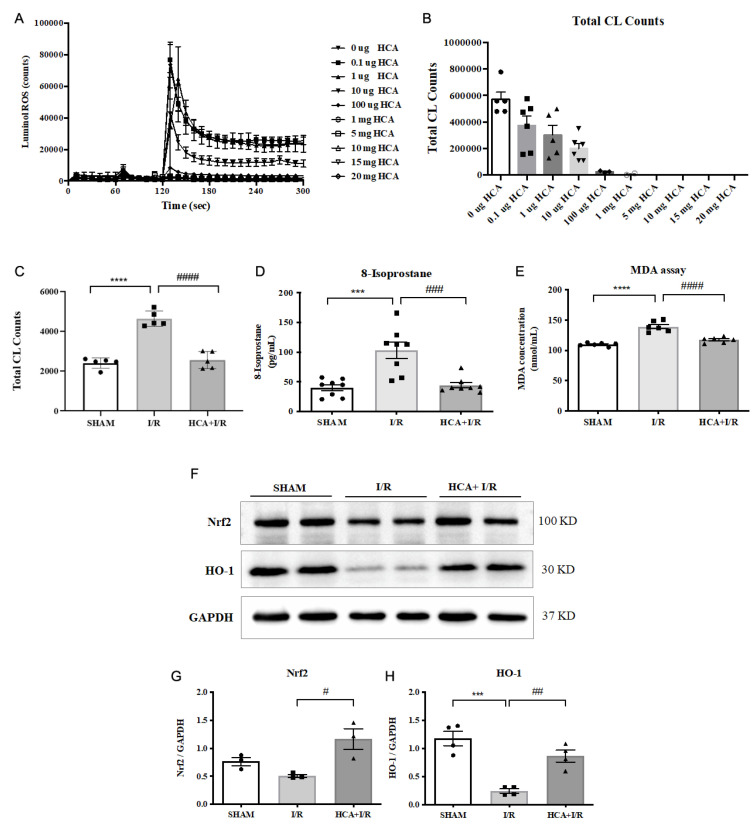
Effect of HCA on antioxidant activity in vitro and in vivo. ROS scavenging activity of HCA in (**A**,**B**) was determined using a CL analyzer. HCA dose-dependently and significantly inhibited ROS activity (**B**). Cardiac I/R significantly increased cardiac ROS (**C**), 8-isoprostane (**D**), and MDA (**E**) vs. sham group, whereas these oxidative stress markers significantly decreased in HCA+I/R vs. I/R group. Original Western blot shows that cardiac I/R decreased cardiac Nrf2 and HO-1 expression in I/R heart (**F**), whereas HCA preconditioning partially restored Nrf2 and HO-1 expression in HCA+I/R heart. The statistical data for Nrf2/GAPDH and HO-1/GAPDH are presented in (**G**,**H**), respectively. All data are expressed as mean ± SEM (n = 3–8). * indicates significant differences vs. sham group (*** *p* < 0.001; **** *p* < 0.0001). # indicates significant differences vs. I/R group (# *p* < 0.05; ## *p* < 0.01; ### *p* < 0.001; #### *p* < 0.0001).

We examined the ROS scavenging activity in vitro and in vivo, as shown in Figure 9. By conducting an analysis with a CL analyzer, it was found that HCA directly, dose-dependently, and significantly suppressed the amount of ROS in vitro (Figure 9A,B). The in vivo data show that cardiac I/R significantly increased the amount of ROS (Figure 9C), 8-isoprostane (Figure 9D), and MDA (Figure 9E) in the I/R group vs. the sham group. HCA preconditioning significantly decreased these oxidative stress markers in the HCA+I/R group vs. the I/R group. An in vivo study using Western blot revealed that cardiac I/R decreased cardiac Nrf2 and HO-1 expression in the I/R heart vs. the sham heart, whereas HCA preconditioning effectively restored cardiac Nrf2 and HO-1 expression in the HCA+I/R heart vs. the I/R heart (Figure 9F). According to the statistical analysis, the ratios of Nrf2/GAPDH (Figure 9G) and HO-1/GAPDH (Figure 9H) significantly decreased in the I/R heart vs. the sham heart; however, the decreased ratios of Nrf2/GAPDH and HO-1/GAPDH significantly recovered in the HCA+I/R heart vs. the I/R heart.

### 2.14. HCA Preconditioning Reduced Cardiac I/R Injury-Induced Expression of Multiple Cytokines

**Figure 10:**
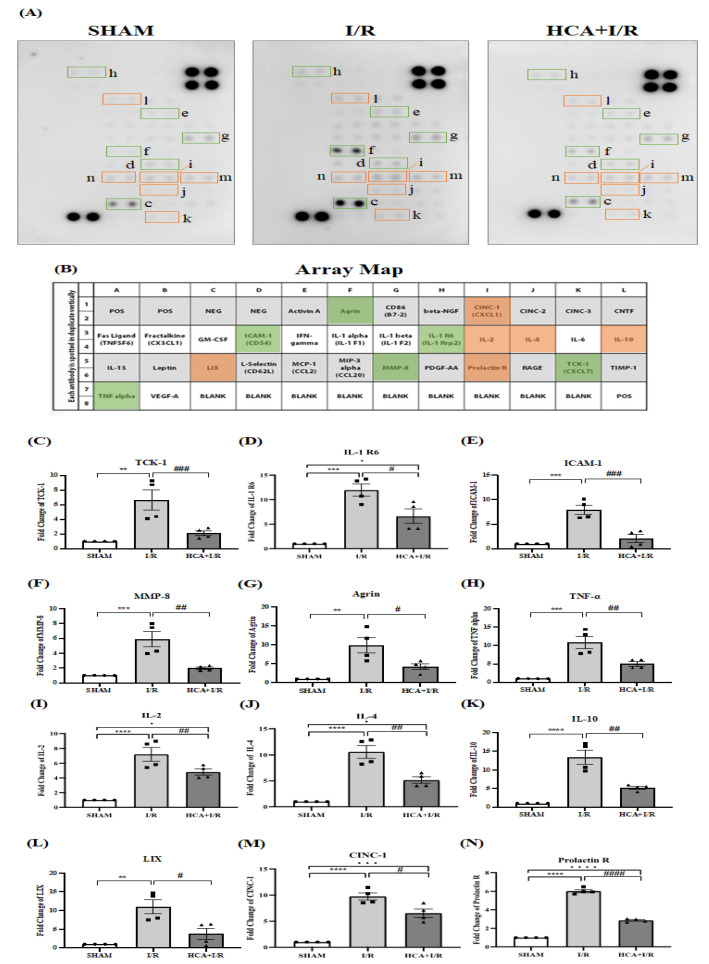
Effect of HCA preconditioning on cardiac I/R injury-induced expression of multiple cytokines in the three groups determined using cytokine array analysis. (**A**) Original cytokine expression. (**B**) Matching dot plot. The expression levels of TCK-1 (**C**), IL-1 R6 (**D**), ICAM-1 (**E**), MMP-8 (**F**), agrin (**G**), TNF-α (**H**), IL-2 (**I**), IL-4 (**J**), IL-10 (**K**), LIX (**L**), CINC-1 (**M**), and prolactin (**N**) are significantly elevated in I/R vs. sham group, whereas they are significantly decreased in HCA+I/R vs. I/R group. All data are expressed as mean ± SEM (n = 4). * indicates significant differences vs. sham group (** *p* < 0.01; *** *p* < 0.001; **** *p* < 0.0001). # indicates the significant differences vs. I/R group (# *p* < 0.05; ## *p* < 0.01; ### *p* < 0.001; #### *p* < 0.0001).

Figure 10A shows the original expression levels of multiple cytokines, with matching dot plots displayed in Figure 10B. The data indicate that the expression levels of TCK-1 (Figure 10C), IL-1 R6 (Figure 10D), ICAM-1 (Figure 10E), MMP-8 (Figure 10F), agrin (Figure 10G), TNF-α (Figure 10H), IL-2 (Figure 10I), IL-4 (Figure 10J), IL-10 (Figure 10K), LIX (Figure 10L), CINC-1 (Figure 10M), and prolactin R (Figure 10N) were significantly elevated in the I/R vs. sham group, whereas they were significantly decreased in the HCA+I/R vs. I/R group.

## 3. Discussion

Our in vitro evidence shows that mimicking cardiac I/R via H_2_O_2_ treatment effectively decreased H9c2 cell viability; however, pretreatment with HCA significantly protected H9c2 cells against H_2_O_2_-induced cell death through the induction of BAG3 upregulation in these cells. Our in vivo data demonstrate that HCA preconditioning significantly restored the decreased cardiac surface blood flow and microcirculation during the I/R periods vs. the I/R group. HCA preconditioning further decreased the increased LVEDP level and restored contractile/relaxing function (±dp/dt level). In an EKG analysis, it was found that HCA preconditioning effectively improved the cardiac I/R-induced increase in ST-segment elevation, P-R interval delay, R-R interval increase, and heart rate decrease. The use of Evans blue-TTC staining also indicated that HCA preconditioning efficiently decreased infarct size vs. the I/R hearts associated with decreased levels of cTn1 and LDH. Regarding the pathologic findings, HCA preconditioning markedly improved cardiac I/R-induced myocardial destruction, erythrocyte extravasation, leukocyte infiltration, tissue edema, and fibrosis vs. the I/R group. According to our data, HCA preconditioning effectively attenuated cardiac I/R-induced oxidative injury and pathologic alterations. In this study, we developed a pharmacologic induction model for the upregulation of cardiac BAG3 and Nrf2/HO-1 signaling to determine the beneficial effect on microvascular dysfunction in cardiac I/R injury. Upregulating BAG3 and Nrf2/HO-1 signaling conferred cardiac and vascular protection against cardiac I/R injury. The microvascular function of cardiac circulation can be effectively reinstituted via HCA preconditioning, providing preventive and palliative benefits for patients with cardiac I/R injury.

Preconditioning with short-term hyperthermia [35], ischemia [36], or pharmacological drugs [29,33] can confer cardiovascular protection against myocardial I/R injury. Our previous report found that thermal preconditioning enhanced cardiac BAG3 expression and inhibited Bax/Bcl-2 ratio-mediated apoptosis formation but did not affect Beclin-1/LC3-II-mediated autophagy in the I/R heart [35]. Cinnamaldehyde has been reported to have many beneficial health-promoting effects, including anti-bacterial [22], anti-cancer [23], anti-inflammation [24], anti-depression [25], and anti-aging [26] effects, as well as providing neurocardiac protection [27,28], possibly through BAG3 upregulation. Cinnamaldehyde treatment can confer protection against several types of cardiovascular diseases, such as viral myocarditis, ischemic heart disease, arterial atherosclerosis, and cardiac hypertrophy [29]. Cardiac I/R significantly decreased cytosolic BAG3 expression in H9c2 cells and the I/R hearts. Our data also confirm that HCA preconditioning indeed upregulates cardiac BAG3 expression, especially in the cytosolic fraction, subsequently leading to cardiac protection. In the present study, the mechanism by which HCA enhanced cytosolic BAG3 overexpression in cardiomyocytes is unclear; thus, further studies are required. BAG3 is an anti-apoptotic co-chaperone protein. A previous study reported that H/R significantly reduced neonatal myocardial cell BAG3 levels, which were associated with enhanced expression of apoptosis markers, decreased expression of autophagy markers, and reduced autophagy flux [14]. Using adenoviral BAG3 gene transfection significantly rescued the I/R-induced decrease in infarct size, improved left ventricular function, and improved autophagy and apoptosis markers [14]. However, ferroptosis, iron-dependent programmed cell death, was observed in our cardiac I/R hearts through a reduction in GPX4 and an increase in 4HNE expression. HCA preconditioning can also ameliorate ferroptotic cell death in cardiac I/R injury associated with decreased peroxidation of lipids, such as 8-isoprostane and MDA.

A delay in the P-R and R-R intervals in the EKG signals and a reduction in heart rate were observed in the cardiac I/R hearts. The delay or block of normal electric transmission in the EKG may be due to the severe structural destruction in the myocytes and coronary artery and the tissue edema in the I/R hearts. The cardiac I/R-induced structural alteration associated with the decrease in BAG3 expression possibly resulted in the electric transmission in the heart. BAG3 overexpression in the heart seems to preserve the structural integrity after cardiac I/R injury and to maintain the EKG parameters. A previous study found that reduced left ventricular ejection fraction and increased left ventricular end-diastolic diameter were associated with BAG3 mutation-related cardiomyopathy, and, in a histologic analysis, myocardial tissue from patients with a BAG3 mutation exhibited myofibril disarray and relocation of the BAG3 protein in the sarcomeric Z-disc [37]. Decreased cardiac expression of BAG3 is correlated with contractile dysfunction and heart failure, decreased BAG3-dependent sarcomere protein turnover impairs mechanical function, and sarcomere force-generating capacity is restored with BAG3 gene therapy [38]. Our data suggest that the cardiac I/R-induced decrease in BAG3 expression was associated with contractile dysfunction, including the increase in LVEDP and decrease in ±dp/dt; however, the overexpression of BAG3 in the cytosolic fraction induced by HCA preconditioning effectively restored these contractile dysfunctions.

BAG3 has been demonstrated to mediate the initiation of the autophagy pathway in HepG2 cells [39]. Tahrir et al. [20] reported that upregulated BAG3 promoted mitochondrial clearance by inhibiting proteasome activity using MG132 to regulate mitochondrial quality control in cardiomyocytes. Our results also revealed that the mitochondrial fission marker DRP1 significantly increased in response to cardiac I/R injury, whereas HCA preconditioning, through the upregulation of BAG3, effectively inhibited DRP 1 expression in the HCA+I/R hearts, indicating a regulatory role of BAG3 in mitochondrial quality control. By triggering the autophagic degradation of impaired mitochondria, mitochondrial fission plays a protective role; however, excessive fission leads to mitochondrial mass loss, ATP deficits, and apoptosis promotion [40]. DRP1 functions with Bax to induce mitochondrial fragmentation, mitochondrial outer membrane permeabilization, and cytochrome C release in response to apoptosis stimulation [41]. In murine and rat models, the inhibition of DRP1 maintains mitochondrial integrity and plays a cardioprotective role during cardiac I/R and cardiac arrest in cells by inhibiting exacerbated fission during I/R injury [40]. A recent report indicated that BAG3 is expressed in primary rat neonatal cardiac fibroblasts and preferentially localizes to mitochondria [42]. The upregulation of BAG3 via HCA preconditioning may localize in the mitochondria, thereby protecting mitochondria against cardiac I/R-induced mitochondrial fission. However, this hypothesis will be examined in the future. In addition, HCA, by directly scavenging ROS activity, according to our data, and by upregulating endogenous antioxidant activity in Nrf2 and HO-1 signaling, provided antioxidant defense against cardiac I/R-induced oxidative stress. In addition, several studies have demonstrated a close interaction between Nrf2 and NF-kB in myocardial I/R injury [43]. Our unpublished figure of NF-kB immunohistochemistry indicates that NF-kB expression was enhanced in the I/R vs. sham control group, whereas the enhanced NF-kB expression was reduced by HCA treatment in the HCA+I/R vs. I/R group.

An I/R-induced inflammatory reaction is one of the most important elements in myocardial I/R injury. Among these, proinflammatory cytokines are a heterogeneous group of mediators that have been associated with the activation of numerous functions, including the immune system and inflammatory responses. The presence of extensive inflammation in the hearts of rats subjected to coronary artery ligation has been previously shown and was confirmed in the present study. Inflammation, determined by the accumulation of inflammatory cells and the measurement of the levels of several cytokines, occurred 4 hr after I/R injury. Importantly, our data demonstrate that HCA pretreatment downregulated inflammatory leukocyte infiltration and the levels of twelve proinflammatory cytokines in the HCA+I/R group vs. the I/R group. These findings provide new insights into the molecular events that resolve inflammation after proinflammatory leukocyte activation in subjects with cardiac I/R injury. The results in Figure 9 show that HCA can inhibit the expression of TCK-1, IL-1 R6, ICAM-1, MMP-8, agrin, TNF-α, IL-2, IL-4, IL-10, LIX, CINC-1, and prolactin and decrease the influx of leukocytes in impaired cardiac tissue.

Because estrogen and sex-specific differences affect the severity of cardiovascular diseases between males and females, we only used male animals in the present study. The reasons for this are as follows: some evidence indicates that sex hormones may exert significant actions on cardiovascular cells between females and males [44]. An ex vivo report suggested that many cardiomyocyte genes are affected in a sex-specific fashion after treatment with estradiol [45]. For example, estradiol treatment led to significantly decreased expression of collagens I and III in female rats, whereas both collagens had increased expression in male rats [46]. Sex differences may affect the tissue level in myocardial diseases [47]. A study demonstrated that sex-specific differences in cardiomyocytes occur before gonads are activated in the embryo [48]. These observations identify that cardiac sex-related differences could occur in cardiovascular diseases [48].

Recently, a report demonstrated its therapeutic effects, molecular mechanisms, and availability in clinical trials [49]. This report indicated cinnamaldehyde’s activity in the treatment of inflammation and cardiovascular diseases [49]. Cinnamaldehyde inhibits the NF-κB pathway and affects inflammatory factors. The natural product provides cardiovascular protection through its anti-inflammatory effect. Preclinical and clinical reports indicate its therapeutic potential and safety in developing new drugs for cardiovascular diseases. Therefore, the clinical relevance and extrapolation of the present in vivo and in vitro results to the human population could be considered in future clinical trials.

In conclusion, HCA preconditioning is an easier strategy for providing cardiac protection against cardiac I/R injury, primarily through the upregulation of the endogenous BAG3-mediated preservation of structural and functional integrity in the damaged heart. HCA also exerts antioxidant activity to scavenge ROS and enhance the Nrf2/HO-1-mediated antioxidant signaling pathway in the heart.

## 4. Materials and Methods

### 4.1. Effect of HCA on In Vitro Model of Cardiac I/R in H9c2 Cells for Determining Viability

H9c2 cells were provided by ATCC (a nonprofit organization that collects, stores, and distributes standard reference cell lines) and plated in 35 mm dishes at a density of 1.0 × 10^6^ cells/dish to reach ~95% confluence. To mimic in vivo cardiac I/R, the cells were treated with 100 μM H_2_O_2_ in regular Ca^2+^ Krebs–Ringer buffer for 12 h, followed by 2 h of recovery in full culture medium. To investigate the effects of HCA, H9c2 cells were pretreated or co-treated with different doses of HCA for 6 h. Cell number was determined using a modified MTT assay (Dojindo Chemicals, Kumamoto, Japan). The pretreatment effect of HCA on BAG3 expression was also determined in H9c2 cells using a Western blot analysis.

### 4.2. Animal Model

A total of 24 male Wistar rats, who were purchased from BioLASCO Taiwan Co., LTD (Ilan, Taiwan) and were 8–10 weeks old (about 250~300 g), were kept at a constant temperature and a fixed photoperiod (light time from 7 am to 6 pm every day) in the Laboratory Animal Center of National Taiwan Normal University. All experiments were approved (approval no: 112058; approval date: 1130131) and followed the guidelines of the National Science Council of the Republic of China (1977) for the care and experimentation of animals. The animals were randomly divided into a sham control group (only threading without ligation), an I/R group, and an HCA+I/R group (n = 8 in each group). HCA pretreatment was administered intraperitoneally (50 mg/kg body weight) three times/week for 2 weeks. At the end of the experiment, the animals were sacrificed via intravenous administration of KCl.

### 4.3. Cardiac I/R Injury Induction

In the cardiac I/R experiment, the rats were first injected with urethane (1.2 g/kg body weight; Sigma, St. Louis, MO, USA), with no significant inhibitory effect observed on the respiratory and circulatory systems. The rat trachea was intubated with an external respirator (Small Animal Ventilator Model 683; Harvard Apparatus, Holliston, MA, USA), which provided a fixed ventilation volume per minute (8 mL/kg) and a positive end-expiratory pressure of 5 cm H_2_O per breath, depending on the anesthetized animal’s respiratory rate at the time, to ensure subsequent respiration of the animal after thoracotomy. Endotracheal intubation was performed by inserting a PE-240 tube between the trachea and C cartilage. Carotid artery cannulation was performed by inserting a PE-50 tube via the left carotid artery into the left ventricle for the measurement of left ventricle pressure, whereas femoral artery cannulation was performed by inserting a PE-50 tube into the left femoral artery for the measurement of arterial blood pressure. Femoral vein cannulation was performed by inserting a PE-50 tube into the right femoral vein to inject normal saline in order to maintain the animals’ physiological body fluids. After the cannulations, the chest was opened to expose the heart, a 6–0 sewing line (6–0 Prolene, UNIK, New Taipei City, Taiwan) was tied to the left anterior descending artery of the coronary artery for 60-min ischemia, and then the knot was loosened for reperfusion for 240 min. In the animals subjected to AMI, AMI was successfully established by a decrease in coronary arterial surface blood flow during ischemia and typical ST-segment elevation on ECG graphics.

### 4.4. EKG Measurement

EKG recording electrodes were inserted subcutaneously in the left and right forelimbs and left hind limbs of the rats using iWorx 214 (IX-214; iWorx Systems, Inc., Dover, NH, USA). Successful establishment of AMI was confirmed using ECG images of typical ST-segment elevation.

### 4.5. Parameters of Left Ventricle Pressure

We measured left ventricular pressure (LVP) parameters, including left ventricular end-diastolic pressure (LVEDP), left ventricular diastolic pressure (LVDP), left ventricular systolic pressure (LVSP), maximum increase in left ventricular pressure (+dp/dt), and maximum decrease in left ventricular pressure (-dp/dt), through the left carotid artery cannulation, as described previously [35].

### 4.6. Cardiac Surface Microcirculation

We determined cardiac surface microcirculation using a MoorFLPI (Moor Instruments, Ltd., Devon, UK). In brief, blood flow on the surface of the heart was continuously monitored using laser speckle imaging and visible light imaging; the principle of this is as follows: Laser Doppler velocity measurement technology is used, and after the laser light source irradiates the surface of the object to be measured, reflected light and scattered light signals are generated, which are captured through the lens. These are analyzed and processed using an algorithm, and a speckle map is obtained. The rendering of the color algorithm is added on the basis of the speckle map, and a color map with different quantitative data is formed. This method is suitable for the detection of continuous intravascular hemoperfusion in tissues. The contrast images are processed to produce 16-color encoded images related to cardiac blood flow, e.g., blue for low flow and red for high flow. The negative control value is set to 0 perfusion units (blue), and the positive control value is set to 1000 perfusion units (red). The perfusion units were analyzed in real time using MoorFLPI V4 software. The successful establishment of an animal model of AMI was verified by a sudden drop in coronary surface blood flow during ischemia.

### 4.7. Infarct Size Calculation

The infarct size in the heart was determined as described previously [20]. Briefly, after reperfusion injury, 5 mL methyl blue was administered via the jugular vein, and then the heart was removed. The heart was serially sliced from the base to the apex at 2 mm intervals. These sections were incubated in 2% 2,3,5-triphenyltetrazolium chloride (TTC, Sigma-Aldrich, St. Louis, MO, USA) at 37 °C for 20 min. The infarct area was stained white, and the area at risk was stained red. Using this method, the area of myocardial infarction, the area at risk, and the total left ventricle (LV) area could be distinguished: blue refers to the normal area, red refers to the ischemia risk area, and white refers to the infarction area. Photographs of the heart slices were taken with a digital camera, and each area was quantified. The ratio of the infarct area to the total area on the scanned image was calculated using ImageJ version 1.54 software: infarct area/area at risk (AAR), AAR/LV, and total infarct area.

### 4.8. Effect of HCA Preconditioning on Cytosolic and Mitochondrial BAG3 Expression

The expression of BAG3 in the cytosol or mitochondria was determined in the sham, HCA preconditioning, I/R, and HCA+I/R hearts. The mitochondrial and cytosolic fractions were obtained using a Mitochondria Isolation Kit for tissue (with Dounce homogenizer, ab110169 (MS8510, Abcam, Cambridge, UK). The protein concentration was determined using a BioRad Protein Assay (BioRad Laboratories, Hercules, CA, USA). Additionally, 10 µg of protein was electrophoresed as described below. The primary antibodies of BAG3 (1:1000, Proteintech, Rosemont, IL, USA) [35], cytochrome C [40] (1:1000, Bioss_bs-0013R), the mitochondrial marker prohibitin (1:1000, Abcam_ab28172), and the cytosolic marker GAPDH (1:5000, Sigma-Aldrich, St. Louis, MO, USA) were used.

### 4.9. Western Blotting

The method used for Western blotting is described in [4]. Frozen left ventricular tissue obtained from the infarcted area of the rat heart was ground and homogenized in liquid nitrogen and dissolved in the buffer of a radio-immunoprecipitation assay (Bio Basic Inc., Markham, ON, Canada) with a protease inhibitor (78430, Thermo Fisher Scientific, Waltham, MA, USA), and the homogenate was centrifuged for 30 min at 14,000 rpm and 4 °C to collect the supernatant. The protein concentrations were determined using a BioRad Protein Assay (BioRad Laboratories). BlueRAY Prestained Protein Ladder (Cat. No. PM006-0500, GeneDireX, Inc., Taoyuan County, Taiwan) was used as a protein standard for protein identification. After electrophoresis and electro-transfer, the blots were blocked using 5% BSA (A7906, Sigma-Aldrich) for 30 min and incubated with primary antibodies overnight at 4 °C. The primary and secondary antibodies used in the Western blot and IHC were as follows: BAG3 (1:1000, Proteintech, Rosemont, USA) [35], LC3II (1:1000, Proteintech, Rosemont, IL, USA) [35], Beclin-1 (1:1000, Proteintech, Rosemont, IL, USA) [35], DRP1 (D6C7, 1:1000, Cell Signaling Technology, Inc., Danvers, MA, USA) [40], β-actin (1:5000, Sigma-Aldrich, Missouri, USA) [35], Nrf2 (EP1808Y1:1000, Abcam) [34], HO-1 (ab13248, 1:1000, Abcam) [34], and GAPDH (1:5000, Sigma-Aldrich, Missouri, USA). Horseradish peroxidase (HRP)-conjugated goat anti-mouse IgG and HRP-conjugated goat anti-rabbit IgG were also used. All protein signals were detected using Western lightning plus-ECL (PerkinElmer, Waltham, MA, USA); imaged using a cold photographic system, namely, an ImageQuestTM LAS-4000 (Fujifilm Co., Tokyo, Japan); and quantified using Image J software.

### 4.10. Antioxidant Activity Determination

An ultrasensitive chemiluminescent analyzer was used to determine the antioxidant activity of HCA, as described previously [50].

### 4.11. Pathologic Findings

After formalin fixation and paraffin embedding, 5 µm sections of the heart samples were subjected to hematoxylin and eosin (H&E) staining and Masson’s trichrome staining.

### 4.12. Immunohistochemistry (IHC)

We performed IHC staining on 10% formalin-fixed and paraffin-embedded cardiac tissue sections. We deparaffinized and rehydrated these sections with xylene and alcohol. After being subjected to antigen retrieval and blocked for non-specific binding, these sections were incubated with primary antibodies overnight at 4 °C. The primary antibodies used were CD45 (ab10558, Abcam, 1:200), BAG3 (1:1000, Proteintech) [35], Beclin-1 (1:1000, Proteintech) [35], and LC3II (1:1000, Proteintech) [35]. These sections were incubated with secondary antibody horseradish peroxidase-labeled polymer (Dako, Glostrup, Denmark) for 1 h at room temperature, immersed in 3′3′-diaminobenzidine, and stained with hematoxylin.

### 4.13. 8-Isoprostane, MDA, and Biochemical Determination

8-Isoprostane in plasma was determined using an 8-Isoprostane ELISA kit (Cayman Chemical, Ann Arbor, MI, USA). MDA was assayed using a Lipid Peroxidation (MDA) Assay Kit (Colorimetric/Fluorometric) (ab118970, Abcam, Cambridge, UK). Caspase 3 activity was determined using a Caspase-3 Assay Kit (ab3941, Colorimetric, Abcam, Cambridge, UK).

### 4.14. Multiple Cytokine Antibody Array

Inflammatory cytokines may contribute to cardiac I/R injury. We therefore determined the expression of cytokines in plasma obtained from the three groups of rats. The expression levels of multiple cytokines were determined using a RayBio^®^rat cytokine Antibody Array C1 (RayBiotech, Inc., Norcross, GA, USA) according to the manufacturer’s instructions.

### 4.15. Statistical Analysis

All data from Image J software are expressed as the mean ± standard error of the mean (SEM). The measures of interest are comparisons of the parameters in the rats that underwent coronary artery ligation with vs. without HCA preconditioning. Differences within groups were evaluated using a paired *t*-test. A one-way analysis of variance was used to establish differences among the groups, followed by Tukey’s post hoc test. Differences were determined to be significant at *p* < 0.05. We used GraphPad Prism 6 (GraphPad Prism 6.0 Software Inc., CA, USA) for graph preparation. All statistical analyses were performed using IBM SPSS Statistics software 29.0.

## 5. Conclusions

In conclusion, HCA preconditioning is an easier strategy for providing cardiac protection against cardiac I/R injury, primarily through the upregulation of the endogenous BAG3-mediated preservation of structural and functional integrity in the damaged heart. HCA also exerts antioxidant activity, scavenging ROS and enhancing the Nrf2/HO-1-mediated antioxidant signaling pathway in the heart.

## Data Availability

The data are confidential.

## Abbreviations

AAR	area at risk
AMI	acute myocardial infarction
BAG3	Bcl-2–associated athanogene
I/R	ischemia/reperfusion
HCA	2′-Hydroxycinnamaldehyde
4HNE	4-hydroxynonenal
HR	heart rate
+dp/dt	maximal rate of rise in left ventricular pressure
-dp/dt	maximal rate of fall in left ventricular pressure
ECG	electrocardiogram
GPX4	glutathione peroxidase 4
HO-1	heme oxygenase-1
HRP	horseradish peroxidase
LAD	left anterior descending coronary artery
LV	left ventricle
LVEDP	left ventricular end-diastolic pressure
LVP	left ventricular pressure
LVSP	left ventricular systolic pressure
MDA	malondialdehyde
Nrf2	nuclear factor erythroid 2–related factor 2
ROS	reactive oxygen species
TTC	2,3,5-triphenyltetrazolium chloride

## References

[1] Luan F., Lei Z., Peng X., Chen L., Peng L., Liu Y., Rao Z., Yang R., Zeng N. (2022). Cardioprotective effect of cinnamaldehyde pretreatment on ischemia/reperfusion injury via inhibiting NLRP3 inflammasome activation and gasdermin D mediated cardiomyocyte pyroptosis. Chem. Interact..

[2] Heusch G. (2020). Myocardial ischaemia–reperfusion injury and cardioprotection in perspective. Nat. Rev. Cardiol..

[3] Hausenloy D.J., Yellon D.M. (2013). Myocardial ischemia-reperfusion injury: A neglected therapeutic target. J. Clin. Investig..

[4] Liu Y., Guo X., Zhang J., Han X., Wang H., Du F., Zeng X., Guo C. (2021). Protective Effects of the Soluble Receptor for Advanced Glycation End-Products on Pyroptosis during Myocardial Ischemia-Reperfusion. Oxid. Med. Cell. Longev..

[5] Shen Y.-C., Lee W.-S., Yang K.-T. (2021). Methyl palmitate protects heart against ischemia/reperfusion-induced injury through G-protein coupled receptor 40-mediated activation of the PI3K/AKT pathway. Eur. J. Pharmacol..

[6] Xu X.N., Jiang Y., Yan L.Y., Yin S.Y., Wang Y.H., Wang S.B., Fang L.H., Du G.H. (2021). Aesculin suppresses the NLRP3 inflam-masome-mediated pyroptosis via the Akt/GSK3β/NF-κB pathway to mitigate myocardial ischemia/reperfusion injury. Phytomedicine.

[7] Liu X.-M., Zhang Z., Zhong J., Li N., Wang T., Wang L., Zhang Q. (2021). Long non-coding RNA MALAT1 modulates myocardial ischemia-reperfusion injury through the PI3K/Akt/eNOS pathway by sponging miRNA-133a-3p to target IGF1R expression. Eur. J. Pharmacol..

[8] Zhang X.-J., Liu X., Hu M., Zhao G.-J., Sun D., Cheng X., Xiang H., Huang Y.-P., Tian R.-F., Shen L.-J. (2021). Pharmacological inhibition of arachidonate 12-lipoxygenase ameliorates myocardial ischemia-reperfusion injury in multiple species. Cell Metab..

[9] Maslov L.N., Popov S.V., Naryzhnaya N.V., Mukhomedzyanov A.V., Kurbatov B.K., Derkachev I.A., Boshchenko A.A., Khaliulin I., Prasad N.R., Singh N. (2022). The regulation of necroptosis and perspectives for the development of new drugs preventing ischemic/reperfusion of cardiac injury. Apoptosis.

[10] Zhang J., Wang Y.T., Miller J.H., Day M.M., Munger J.C., Brookes P.S. (2018). Accumulation of Succinate in Cardiac Ischemia Primarily Occurs via Canonical Krebs Cycle Activity. Cell Rep..

[11] Gottlieb R.A., Bernstein D. (2016). Mitochondrial remodeling: Rearranging, recycling, and reprogramming. Cell Calcium.

[12] Daiber A., Hahad O., Andreadou I., Steven S., Daub S., Münzel T. (2021). Redox-related biomarkers in human cardiovascular disease-classical footprints and beyond. Redox Biol..

[13] Yu C., Xiao J.-H. (2021). The Keap1-Nrf2 System: A Mediator between Oxidative Stress and Aging. Oxidative Med. Cell. Longev..

[14] Su F., Myers V.D., Knezevic T., Wang J., Gao E., Madesh M., Tahrir F.G., Gupta M.K., Gordon J., Rabinowitz J. (2016). Bcl-2-associated athanogene 3 protects the heart from ische-mia/reperfusion injury. JCI Insight.

[15] Knezevic T., Myers V.D., Gordon J., Tilley D.G., Sharp T.E., Wang J., Khalili K., Cheung J.Y., Feldman A.M. (2015). BAG3: A new player in the heart failure paradigm. Heart Fail Rev..

[16] Homma S., Iwasaki M., Shelton G.D., Engvall E., Reed J.C., Takayama S. (2006). BAG3 Deficiency Results in Fulminant Myopathy and Early Lethality. Am. J. Pathol..

[17] Jackson S., Schaefer J., Meinhardt M., Reichmann H. (2015). Mitochondrial abnormalities in the myofibrillar myopathies. Eur. J. Neurol..

[18] Mizushima W., Sadoshima J. (2017). BAG3 plays a central role in proteostasis in the heart. J. Clin. Investig..

[19] Carrizzo A., Damato A., Ambrosio M., Falco A., Rosati A., Capunzo M., Madonna M., Turco M.C., Januzzi J.L., De Laurenzi V. (2016). The prosurvival protein BAG3: A new participant in vascular homeostasis. Cell Death Dis..

[20] Tahrir F.G., Knezevic T., Gupta M.K., Gordon J., Cheung J.Y., Feldman A.M., Khalili K. (2016). Evidence for the Role of BAG3 in Mitochondrial Quality Control in Cardiomyocytes. J. Cell. Physiol..

[21] Dodson M., Castro-Portuguez R., Zhang D.D. (2019). NRF2 plays a critical role in mitigating lipid peroxidation and ferroptosis. Redox Biol..

[22] Sun Q., Li J., Sun Y., Chen Q., Zhang L., Le T. (2020). The antifungal effects of cinnamaldehyde against Aspergillus niger and its application in bread preservation. Food Chem..

[23] Kim T.W. (2021). Cinnamaldehyde induces autophagy-mediated cell death through ER stress and epigenetic modification in gastric cancer cells. Acta Pharmacol. Sin..

[24] Liu P., Wang J., Wen W., Pan T., Chen H., Fu Y., Wang F., Huang J.H., Xu S. (2020). Cinnamaldehyde suppresses NLRP3 derived IL-1β via activating succinate/HIF-1 in rheumatoid arthritis rats. Int. Immunopharmacol..

[25] Gao Z.Y., Chen T.Y., Yu T.T., Zhang L.P., Zhao S.J., Gu X.Y., Pan Y., Kong L.D. (2022). Cinnamaldehyde prevents intergenera-tional effect of paternal depression in mice via regulating GR/miR-190b/BDNF pathway. Acta Pharmacol. Sin..

[26] Pang D., Huang Z., Li Q., Wang E., Liao S., Li E., Zou Y., Wang W. (2021). Antibacterial Mechanism of Cinnamaldehyde: Modu-lation of Biosynthesis of Phosphatidylethanolamine and Phosphatidylglycerol in *Staphylococcus aureus* and *Escherichia coli*.. J. Agric. Food Chem..

[27] Bektaşoğlu P.K., Koyuncuoğlu T., Demir D., Sucu G., Akakın D., Eyüboğlu I.P., Yüksel M., Çelikoğlu E., Yeğen B., Gürer B. (2021). Neuroprotective Effect of Cinnamaldehyde on Secondary Brain Injury After Traumatic Brain Injury in a Rat Model. World Neurosurg..

[28] Tian J., Shan X.-L., Wang S.-N., Chen H.-H., Zhao P., Qian D.-D., Xu M., Guo W., Zhang C., Lu R. (2021). Trans-cinnamaldehyde suppresses microtubule detyrosination and alleviates cardiac hypertrophy. Eur. J. Pharmacol..

[29] Lan H., Zheng Q., Wang K., Li C., Xiong T., Shi J., Dong N. (2023). Cinnamaldehyde protects donor heart from cold ische-mia-reperfusion injury via the PI3K/AKT/mTOR pathway. Biomed. Pharmacother..

[30] Lee C., Hong D., Han S., Park S., Kim H., Kwon B.-M., Kim H. (1999). Inhibition of Human Tumor Growth by 2′-Hydroxy- and 2′-Benzoyl-oxycinnamaldehydes. Planta Medica.

[31] Hong S., Ismail I.A., Kang S., Han D.C., Kwon B. (2016). Cinnamaldehydes in Cancer Chemotherapy. Phytother. Res..

[32] Nguyen H.-A., Kim S.-A. (2016). 2′-Hydroxycinnamaldehyde induces apoptosis through HSF1-mediated BAG3 expression. Int. J. Oncol..

[33] Song F., Li H., Sun J., Wang S. (2013). Protective effects of cinnamic acid and cinnamic aldehyde on isoproterenol-induced acute myocardial ischemia in rats. J. Ethnopharmacol..

[34] Kim N., Trinh N., Ahn S., Kim S. (2020). Cinnamaldehyde protects against oxidative stress and inhibits the TNF-α-induced inflammatory response in human umbilical vein endothelial cells. Int. J. Mol. Med..

[35] Chen Y.H., Chiang C.Y., Chang T.C., Chien C.T. (2020). Multiple Progressive Thermopreconditioning Improves Cardiac Ische-mia/Reperfusion-induced Left Ventricular Contractile Dysfunction and Structural Abnormality in Rat. Transplantation.

[36] Murry C.E., Jennings R.B., Reimer K.A. (1986). Preconditioning with ischemia: A delay of lethal cell injury in ischemic myocardium. Circulation.

[37] Domínguez F., Cuenca S., Bilińska Z., Toro R., Villard E., Barriales-Villa R., Ochoa J.P., Asselbergs F., Sammani A., Garcia-Pavia P. (2018). Dilated Cardiomyopathy Due to BLC2-Associated Athanogene 3 (BAG3) Muta-tions. J. Am. Coll. Cardiol..

[38] Martin T.G., Myers V.D., Dubey P., Dubey S., Perez E., Moravec C.S., Willis M.S., Feldman A.M., Kirk J.A. (2021). Cardiomyo-cyte contractile impairment in heart failure results from reduced BAG3-mediated sarcomeric protein turnover. Nat. Commun..

[39] Liu B.-Q., Du Z.-X., Zong Z.-H., Li C., Li N., Zhang Q., Kong D.-H., Wang H.-Q. (2013). BAG3-dependent noncanonical autophagy induced by proteasome inhibition in HepG2 cells. Autophagy.

[40] Tahrir F.G., Langford D., Amini S., Mohseni Ahooyi T., Khalili K. (2019). Mitochondrial quality control in cardiac cells: Mecha-nisms and role in cardiac cell injury and disease. J. Cell. Physiol..

[41] Große L., Wurm C.A., Brüser C., Neumann D., Jans D.C., Jakobs S. (2016). Bax assembles into large ring-like structures remodel-ing the mitochondrial outer membrane in apoptosis. EMBO J..

[42] Martin T.G., Sherer L.A., Kirk J.A. (2024). BAG3 localizes to mitochondria in cardiac fibroblasts and regulates mitophagy. Am. J. Physiol. Circ. Physiol..

[43] Hwa J.S., Jin Y.C., Lee Y.S., Ko Y.S., Kim Y.M., Shi L.Y., Kim H.J., Lee J.H., Ngoc T.M., Bae K.H. (2012). 2-methoxycinnamaldehyde from Cinnamomum cassia reduces rat myocardial ischemia and reperfusion injury in vivo due to HO-1 induction. J. Ethnopharmacol..

[44] Regitz-Zagrosek V., Gebhard C. (2023). Gender medicine: Effects of sex and gender on cardiovascular disease manifestation and outcomes. Nat. Rev. Cardiol..

[45] Kararigas G., Bito V., Tinel H., Becher E., Baczko I., Knosalla C., Albrecht-Küpper B., Sipido K.R., Regitz-Zagrosek V. (2012). Transcriptome characterization of estrogen-treated human myocardium identifies myosin regulatory light chain interacting protein as a sex-specific element influencing contractile function. J. Am. Coll. Cardiol..

[46] Dworatzek E., Mahmoodzadeh S., Schriever C., Kusumoto K., Kramer L., Santos G., Fliegner D., Leung Y.K., Ho S.M., Zimmermann W.H. (2019). Sex-specific regulation of collagen I and III expression by 17β-estradiol in cardiac fibroblasts: Role of estrogen receptors. Cardiovasc. Res..

[47] Regitz-Zagrosek V., Kararigas G. (2017). Mechanistic pathways of sex differences in cardiovascular disease. Physiol. Rev..

[48] Shi W., Sheng X., Dorr K.M., Hutton J.E., Emerson J.I., Davies H.A., Andrade T.D., Wasson L.K., Greco T.M., Hashimoto Y. (2021). Cardiac proteomics reveals sex chromosome-dependent differences between males and females that arise prior to gonad formation. Dev. Cell.

[49] Guo J., Yan S., Jiang X., Su Z., Zhang F., Xie J., Hao E., Yao C. (2024). Advances in pharmacological effects and mechanism of action of cinnamaldehyde. Front Pharmacol..

[50] Chien C.Y., Chien C.T., Wang S.S. (2014). Progressive thermopreconditioning attenuates rat cardiac ischemia/reperfusion injury by mitochondria-mediated antioxidant and antiapoptotic mechanisms. J. Thorac. Cardiovasc. Surg..

